# Heat-stimuli-enhanced osteogenesis using clinically available biomaterials

**DOI:** 10.1371/journal.pone.0181404

**Published:** 2017-07-18

**Authors:** Takehiro Ota, Yoshihiro Nishida, Kunihiro Ikuta, Ryuji Kato, Eiji Kozawa, Shunsuke Hamada, Tomohisa Sakai, Naoki Ishiguro

**Affiliations:** 1 Department of Orthopaedic Surgery, Nagoya University Graduate School and School of Medicine, Nagoya, Japan; 2 Department of Basic Medical Sciences, Graduate School of Pharmaceutical Sciences, Nagoya University, Nagoya, Japan; University of Texas at San Antonio, UNITED STATES

## Abstract

A recent study reported that heat stress stimulates osteogenesis in an in vivo rat model using alginate gel and magnetite cationic liposomes. However, for clinical use, the efficacy for promoting osteogenesis needs to be investigated using clinically approved materials, and preferably with animals larger than rats. The aim of this study was to evaluate multiple heat stimuli-triggered osteogenesis in rat tibial defect models using already clinically applicable materials (Resovist® and REGENOS®) and determine the efficacy also in the rabbit. Fifty-eight rats and 10 rabbits were divided into two groups, respectively, with or without hyperthermia treatment at 45°C for 15 min. (hyperthermia; 20 rats once a week, 8 rats three times a week, 5 rabbits once a week, control; 30 rats and 5 rabbits). Micro-CT assessment at 4 weeks revealed that a significantly stimulated osteogenesis was observed in the once a week group of both rats and rabbits as compared to the control group (p = 0.018 and 0.036, respectively). In contrast, the three times a week group did not show enhanced osteogenesis. Histological examination and image analysis showed consistent results in which the area of mineralized bone formation in the once a week hyperthermia group was significantly increased compared with that in the control group at four weeks (rat; p = 0.026, rabbit; p = 0.031). Newly formed bone was observed in the grafted materials from the periphery toward the center, and more osteoclasts were found in the once a week group. Heat stress also induced enhanced alkaline phosphatase expression in cultured osteoblastic cells, MC3T3, in vitro (p = 0.03). On the other hand, heat stress had no obvious effects on chondrogenic differentiation using ATDC5 cells. Our study demonstrates that heat-stimuli with clinically applicable novel heating materials can promote significant osteogenesis, and may thus be a promising treatment option for diseases associated with bone defects.

## Introduction

Patients with bone defects after resection of bone tumors, complicated fractures, and chronic infections should be adequately treated by specialist physicians. Autologous bone grafts have long been used because of their osteoinductive activity and subsequent osteogenesis [1,2]. However, the collection of sufficient autologous bone is occasionally difficult, particularly in children and the elderly. There are concerns about complications at the donor site, such as infection, pain, and paresthesia. There is some dispute over the implementation of such invasive interventions to compensate for defects, particularly in debilitated patients. Although allograft bone has similar properties to those of autologous bone and is widely used, particularly in western countries, this does not remove risks such as infection, and since there exists no system for organizing the provision of allogenic bone in eastern countries mainly due to religious issues, it is not currently a realistic option. Because of that, a variety of bone substitutes such as tricalcium phosphate and hydroxyapatite (HA), have been introduced to overcome and/or minimize these disadvantages [3,4], but they have the disadvantage of no or insufficient capacity of osteoinductive ability compared with autologous bone grafts. Consequently, there is demand for the development of more effective methods for the promotion of osteogenesis.

Hyperthermia is a therapy being investigated for inflammatory disease [5,6] and metastatic disease [7,8]. Various methods for implementing hyperthermia have been reported previously, including the use of warm water [9], induction heating [10], and radio waves [11]. The main challenge for hyperthermia is to apply an appropriate heat to only the target region. One clinical problem is how to apply heat to the target site to minimize the influence on the surrounding, normal tissue and prevent complications. One reported strategy for solving this problem is to induce hyperthermia by using alternating magnetic fields (AMF) with iron particles [12–15]. Hyperthermia is based on the principle that a magnetic particle can generate heat by hysteresis loss under an AMF. Using magnetite nanoparticles, we could achieve artificial local control of temperature within selected region.

Among such research into hyperthermia, several old reports have noted that heat stress promotes bone growth [16–18]. Several other studies using human mesenchymal stem cells (MSCs), bone marrow stromal cells and MG63, demonstrated that differentiation or proliferation of osteoblasts, alkaline phosphatase (ALP) activity, and osteogenic markers were elevated in response to heat stress in vitro [19–21]. However, many aspects of the mechanism by which heat induces osteogenesis remain elusive. Olkku et al. have suggested that Wnt signaling may be activated in response to US-induced temperature rise [22], and some studies have shown the possibility that elevations in heat shock protein (HSP) contribute to the promotion of osteogenesis [21,23–25]. On the other hand, there are reports that hyperthermia inhibits osteogenesis. Dolan et al. reported that osteoblast-like MC3T3 cells exposed to severe heat shock over 47°C for 30 sec resulted in cell necrosis and apoptosis, although damage levels were less than those of osteocytes, and MSCs were stimulated to induce osteogenesis [26]. Referring to other past reports [27,28], it is likely that cell and tissue damage will occur at temperatures of 47–48°C.

However, reports focusing on in vivo osteogenesis are rare. Previously, we reported on the osteogenesis-promoting effect of heating by AMF using magnetite cationic liposomes (MCL) in rats [29]. In that study, the rats in the hyperthermia group exhibited significant osteogenesis compared to the control group, with no obvious side effects from heating between 43–46°C for 15 minutes. That was the first report of implementation in an in vivo animal model and suggested the possibility that heat stimulation could be used as a new treatment modality for bone defects.

In this study, we evaluated heat stimuli-triggered osteogenesis in rat and rabbit models using already clinically applicable materials instead of MCL and alginate. We also examined the effect of hyperthermia in two cell lines, osteoblast-like MC3T3 cells and ATDC5, the latter of which shows chondrogenic to osteogenic differentiation.

## Material and method

### Preparation of heating materials

**Fig 1:**
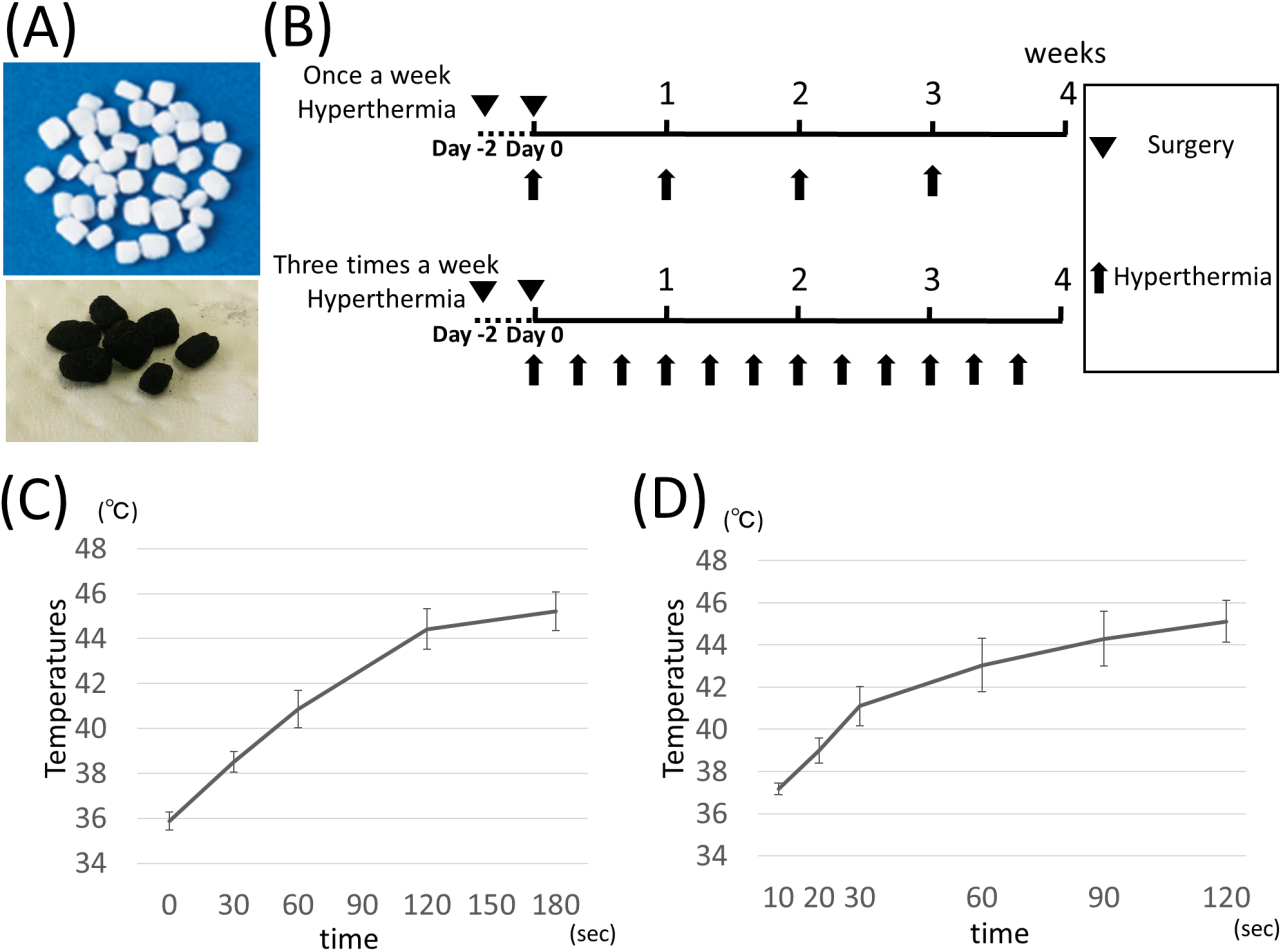
Experimental protocol of hyperthermia and efficacy of hyperthermia. (A) Heating materials. Ferucarbotran was absorbed into HA under negative pressure. HA (upper figure), composites (lower figure). (B) Scheme of the hyperthermia protocol (Triangles; surgical procedures, arrows; hyperthermia treatment). Hyperthermia treatment was accurately applied at 45°C for 15 min by adjusting the magnetic field intensity. Temperatures were measured at the surface of the material with thermofiber probe (C; rat, D; rabbit). The desired temperature could be achieved by adjusting the magnetic field intensity.

Heating materials were made of iron Magnetic resonance imaging (MRI) contrast agent for liver, Ferucarbotran (Resovist® FUJIFILM RI Pharma, Tokyo, Japan), and HA scaffold (REGENOS® Kuraray, Osaka, Japan). Resovist® is a hydrophilic colloid of the superparamagnetic iron oxide nanoparticles used for contrast media of MRI and it can be heated by AMF [30]. Resovist® includes 540mg Ferucarbotran (0.5mmol Fe/ml, 27.9mg iron) per 1ml as a stable colloid solution. Ferucarbotran core size is 3-5nm, and hydrodynamic diameter is 57-59nm. Their crystal structure is compatible with γ- Fe_2_O_3_ and magnetic susceptibility is 107emu/g Fe [31]. We concentrated Resovist® of 1ml to 150μl to obtain sufficient thermal energy. REGENOS® is a HA characterized by a superior penetration with the unidirectional porous structures, and this structure had strong capillary action absorbing Resovist® completely. In order to stabilize absorbed Resovist® in REGENOS®, we mixed the two materials under negative pressure. This concentrated Resovist® (150μl, 27.9mg iron contained) was mixed with ten granular REGENOS® (10mg each), and one granular REGENOS® (including 2.79mg iron) was used for one rat tibial defect. The concentrated Resovist® was also mixed with two cylindrical REGENOS® (8 mm diameter × 4 mm height: 50mg each), and one cylindrical REGENOS® (including 14mg iron) was used for one rabbit tibial defect. REGENOS® and heating composites are shown in Fig 1A.

### Animals and surgical procedures

The experimental protocol was approved by the Animal Care Committee of our institution (approved number: 29366) and the experiments were performed according to the principles outlined in the US National Academy of Sciences' Guide for the Care and Use of Laboratory Animals. Each animal had free access to food and water, and housed in a separate cage with a 12/12 light/dark cycle. All animals were housed in a temperature at 23°C± 2°C for rats and 21°C± 2°C for rabbits. The condition of the animals was monitored every day. To evaluate thermal effects on bone formation, we established a tibial defect model in rats and rabbits according to the procedures described in previous studies [29,32,33]. Fifty-eight male 8-week-old Sprague-Dawley rats weighing approximately 250g were used in the experiments. Each rat was anesthetized with an inhalation of isoflurane followed by an intraperitoneal injection of sodium pentobarbital (50mg/kg). The proximal site of the right tibia was exposed after skin incision, and the periosteum was retracted to expose cortical bone surface. A circular unicortical defect of 3mm in diameter was created on the anteromedial surface of the proximal metaphysis using a burr drill. The overlying muscle and skin were closed in layers. Ten adult male New Zealand white rabbits weighing 2.5–3.0 kg were anesthetized with an inhalation of isoflurane followed by an intramuscular injection of Ketamine (30mg/kg) and xylazine (2mg/kg). A circular unicortical defect of 8mm in diameter was created on the anteromedial surface at the proximal metaphysis using a burr drill. Then, the overlying muscle and skin were closed in layers. Two days after the initial surgery, the defects were exposed, and combination of heating materials or HA only was transplanted and stabilized in the defects. In both rats and rabbits, the circular unicortical defect was designed to fit the size of grafted materials. To avoid protrusion of the materials, overlying fascia was firmly sutured. Rats and rabbits were divided into a hyperthermia group (28 rats and 5 rabbits), three control groups: control 1; heating composite (HA + Ferucarbotran) (+) without AMF (20 rats and 5 rabbits), control 2; HA (+) with AMF (5 rats), control 3; HA (+) without AMF (5 rats). The rat hyperthermia group was sub-divided into a once a week hyperthermia group (n = 20) and three times a week hyperthermia group (n = 8) to examine the effects of hyperthermia frequency on osteogenesis. Each group was observed until 4 weeks after the start of hyperthermia. At the termination of experiments, each rat was euthanized by an intraperitoneal injection of overdose sodium pentobarbital (150mg/kg) under inhalation of 3.5% isoflurane, and each rabbit was euthanized by using an intra-cardiac injection of 150–200 mg/kg sodium pentobarbital after intramuscular injection of Ketamine (30mg/kg) and xylazine (2mg/kg). All efforts were exerted to minimize suffering.

### Hyperthermia treatment

The hyperthermia group and control 2 were subjected to AMF immediately after implantation of heating materials or HAs as described in the previous study [29]. The control 1 (both rat and rabbit experiments) and 3 group were implanted with heating materials or HAs, respectively, and were not treated with AMF. An AMF was generated by a vertical coil with an inner diameter of 7 cm, driven by a transistor inverter (LTG-100-05, Dai-ichi High Frequency, Tokyo) at a frequency of 118KHz. The applied temperature was adjusted to 45°C which was proven effective for osteogenesis in the previous study [29]. As the temperature at the surfaces of the materials was measured through the open wound with FX-9020 optical fiber probes (Anritsu Meter, Kyoto, Japan), hyperthermia was accurately applied at 45°C for 15 min by adjusting the magnetic field intensity. The same intensity was used for further hyperthermia treatment. Temperature did not rise over 40°C in control 2 group under AMF for 15 min. Hyperthermia treatment was applied as shown in Fig 1B.

### Micro-computed tomography (micro-CT) analysis

Effects of hyperthermia on osteogenesis were evaluated radiographically. The right tibias of rats and rabbits were scanned with a micro-computed tomography (micro-CT) scanner (Skyscan-1176; Bruker, Belgium). Images were captured every 0.5 degrees through 180 degrees of the bone at 65kV and 385 μA, with a 1.0 aluminum filter. Image reconstruction using the Skyscan NRecon software (Bruker, Belgium) provided an isotropic image pixel size of 18μm. Based on the results of the preliminary experiments that new bone formation was observed in the heating materials of the rat model, and in and around the heating materials in the rabbit model, the regions of interest (ROI) were defined differently between rats and rabbits. Consolidation area in the transplanted heating material was defined as ROI in rats, and that in and adjacent to the heating materials (5mm around the materials) in rabbit models. The newly formed bone volume (BV) was measured using Skyscan CTAn software. Because HA is a radiopaque material, newly formed bone was defined as an area of increased CT value as compared to that of HA only. The bone mineral density (BMD) of HA was also measured in the rat model to normalize that of HA plus newly formed bone. We evaluated the effects of hyperthermia on bone formation at 2 and 4 weeks after implantation in the rat model, and at 4 weeks after implantation in the rabbit model.

### Villanueva-Goldner staining

New bone formation with or without hyperthermia was evaluated with Villanueva-Goldner bone staining. At 2 and 4 weeks after the initial treatment for rats (control 1 and hyperthermia; four rats each), and 4 weeks for rabbits (control 1 and hyperthermia; three rabbits each), right tibia was excised, and fixed with 70% ethanol for 3days, dehydrated through graded ethanol series and embedded in methyl methacrylate without decalcification (Tohkai Cytopathology Institute, Gifu, Japan). The embedded tissues were cut into 6-μm-thick sagittal sections and evaluated under a light microscope. The most central and maximally sectioned surface of the tibial bone defect and transplanted heating material was subjected to histomorphometric analysis. Images of prepared slides were captured with a digital camera (DP71, Olympus, Japan) under a light microscope (BX60, Olympus, Japan). Mineralized bone tissues are dyed in green and nonmineralized osteoid tissues in red by Villanueva-Goldner stain. The newly formed bone (green area) was marked and subjected to quantification with Image J software. The amount of the newly formed bone area was normalized by the area of the posterior cortex where the surgical procedures and hyperthermia had a minimal effect, as previously described [29].

### ALP/TRAP staining

Rat tibial bone at 4 weeks after implantation was excised, and fixed with 90% ethanol for 1 week. After dehydration, the specimens were embedded in glycidyl methacrylate (GMA) without decalcification (Tohkai Cytopathology Institute, Gifu, Japan). The embedded tissues were cut into 6-μm-thick sagittal sections. The most central and maximally sectioned surface of each tibia and heating material was subjected to histological examination. The bone formation and bone resorption activity were determined by double staining of tartaric acid-resistant phosphatase (TRAP) using sodium tartrate (Kishida Chemical, Osaka, Japan) and sodium naphthol AS-B1 phosphate (Sigma-Aldrich, St. Louis, MO, USA), and alkaline phosphatase (ALP)by using naphthol AS-MX phosphate (Wako Pure Chemical, Osaka, Japan) and Fast Blue RR Salt (Sigma). Images of prepared slides were captured with a digital camera (DP71, Olympus, Japan) under the light microscope (BX60, Olympus, Japan). TRAP-positive cells adjacent to the HA or newly formed bone were estimated as osteoclasts, and ALP-positive cells adjacent to the newly formed bone were estimated as osteoblasts. The numbers of TRAP-positive cells were counted in randomly selected 5 different areas under a light microscope at 100x magnification. Mean numbers of positively stained cells were calculated.

### Effects of hyperthermia in vitro

Two cell lines, MC3T3 and ATDC5, were obtained from RIKEN Cell Bank (Tsukuba Science City, Japan), and used to investigate the effects of hyperthermia on osteoblastic differentiation and endochondral ossification, respectively. The osteoblastic cell line, MC3T3 cells, was maintained with in α-MEM (Gibco BRL, Grand Island, NY), supplemented with 10% fetal bovine serum, 1% penicillin/streptomycin solution. To induce osteoblastic differentiation, 50 μg/ml of ascorbic acid, 10–6 M of dexamethasone and 10mM of beta-glycerophosphate were added and cultured at 37°C with 5% CO2 in the air. The medium was replaced every 2 days. The mouse chondrogenic cell line, ATDC5, was maintained in a 1:1 mixture of Dulbecco’s modified Eagle’s medium and Ham’s F-12 medium (Sigma) containing 5% fetal bovine serum and antibiotics (penicillin: 100 U/ml, streptomycin: 100 μg/ml, amphotericin-B: 0.25 μg/ml) at 37°C under 5% CO2 in air. ITS Liquid Media Supplement (Sigma) was added to the medium at a concentration of 10 μg/ml to induce chondrogenic differentiation. The medium was replaced every 2 days. Cells were seeded in 6-well plates at a density of 5 × 10^5^ cells per well, and preincubated at 37°C for 48 h with condition medium. Hyperthermia was performed using a thermostatic water bath controlled to an accuracy of 1°C. After a single exposure to various temperatures (control; 37°C, hyperthermia; 41, 43 and 45°C) for 15 minutes, the medium was removed and substituted with fresh differentiation medium to avoid any possible loss of water or contained materials caused by hyperthermia. MC3T3 and ATDC5 cells were cultured for up to 28 days to observe the process of differentiation. To evaluate the osteoblastic differentiation of treated MC3T3, ALP expression was determined by immunocytochemistry. MC3T3 cells were fixed with 45% acetone, 10% ethanol containing citrate buffer pH 5.4 and stained using substrate for ALP (Takara Bio, Inc.) according to the manufacturer's instructions. Expression of ALP was determined with this staining every 7days (day 7, day 14, and day 21). For semi-quantitative analyses of ALP expression, all stained areas in the wells were scanned and measured with Image J software (National Institutes of Health, Bethesda, MD). To evaluate the effects of hyperthermia on the endochondral ossification of ATDC5 cells, the deposition of sulfated glycosaminoglycans was determined with Alcian blue staining. ATDC5 were fixed with 100% methanol, and stained with 0.1% Alcian blue 8GS (Sigma) in 0.1 N HCl for 4 h at room temperature. Deposition of the proteoglycan-rich matrix was determined with this staining every 7 days (day 7, day 14, and day 21). For quantitative analyses of Alcian blue-positive area, the dye was extracted with 6 M guanidine HCl overnight, and the total optical density of the extracted dye was measured using a spectrophotometer at 620 nm. Cell viability after hyperthermia was analyzed with MTS proliferation assay. Cells were seeded in 96-well plates at a density of 1 x 10^5^ cells per well and preincubated at 37°C for 48 h with condition medium. After the exposure to various temperatures (37–45°C) for 15 minutes, cell viability was evaluated at day 7, 14, and 21.

### Statistical analysis

Hyperthermia and control differences between values for each bone volume were assumed as 0.2 based on the preliminary experiments, and each group was normally distributed with standard deviation 0.1. Under the hypothesis that means of the hyperthermia and control groups are equal with probability (power) 0.8, the Type I error probability associated with this test is 0.05. Based on the assumption, we needed to study at least 5 subjects of hyperthermia and control groups each. Comparisons between two groups were analyzed with Student's t-test and P values < 0.05 were considered significant. Statistical analysis was performed using SPSS™ software, version 23.0 (Chicago, IL). All results were presented as mean ± standard deviation (SD).

## Results

### Efficacy of warming effects in rat and rabbit tibial defect model

Before examining the effects of hyperthermia on osteogenesis, the warming effects of the heating composite, HA, and Ferucarbotran, which have been approved for clinical use, need to be determined. Temperatures of the surface of the heating material at the tibial defects were measured with optical fiber probes which were described previously [29]. In brief, the surface of the heating material reached 45°C in 3 min for rat and 2 min for rabbit (Fig 1C and 1D, respectively). The temperature could be maintained at 45°C for 15 min by adjusting the AMF power. Temperatures of surrounding soft tissues were measured and confirmed under 41°C in both models. No rats or rabbits suffered skin burns or other recognizable complications during the treatment period.

### Micro-CT analyses

**Fig 2:**
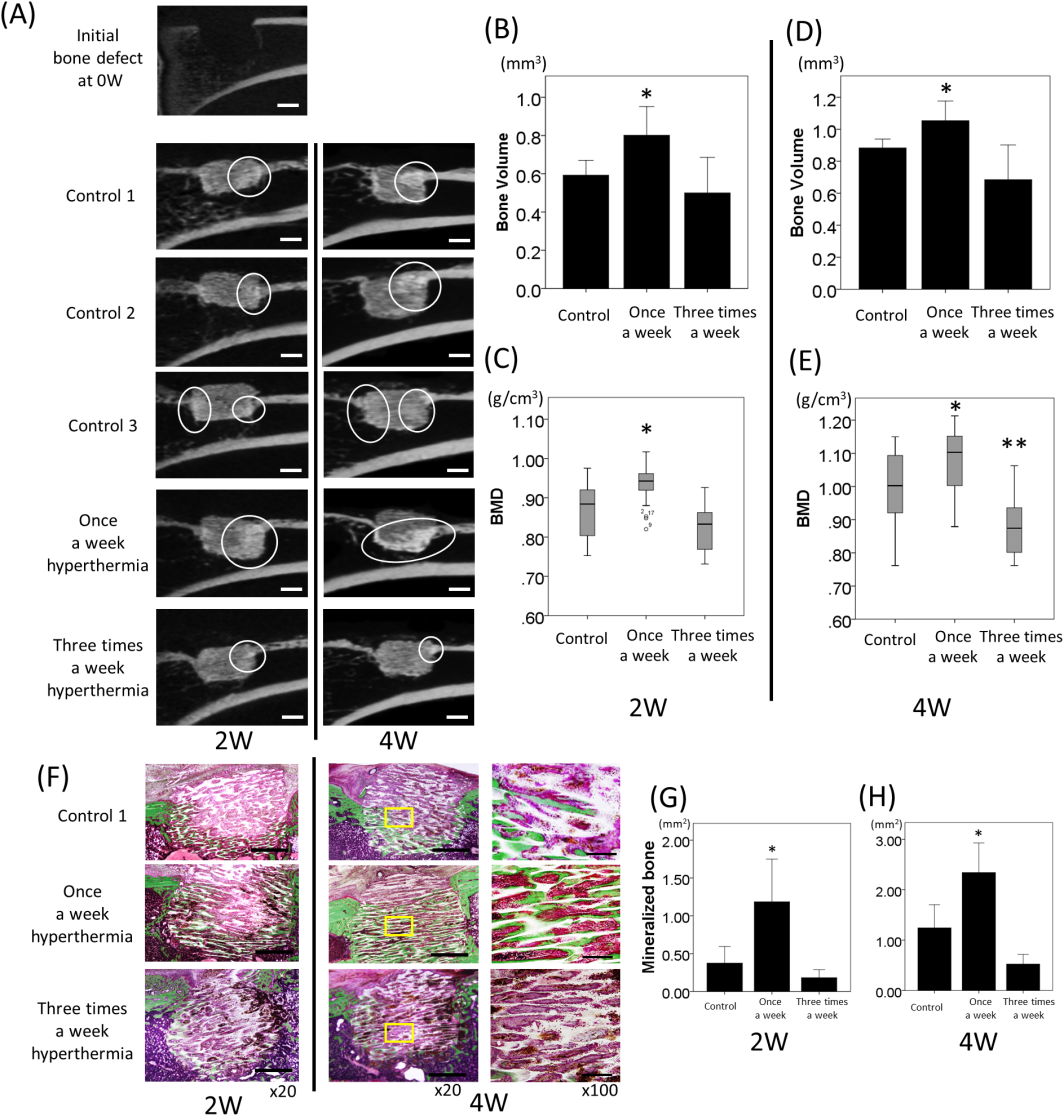
Radiographic and histological evaluation of newly formed bone with hyperthermia using a rat model. (A) Sagittal micro-CT image of the rat right tibial defect, control 1, 2, and 3, once a week, three times a week hyperthermia groups at 2 weeks and 4 weeks (bars; 1mm). White circling depicts newly formed bone at each time point. Measured bone volume (B) and BMD (C) in the rat models were graphed (control includes control 1, 2, and 3; n = 30, once a week hyperthermia; n = 20, three times a week; n = 8) at 2 weeks. Bone volume (D) and BMD (E) in the rat models (control; n = 26, once a week hyperthermia; n = 16, three times a week; n = 4,) were graphed at 4 weeks. *p<0.05 compared with control group. **p<0.05 compared with control and once a week hyperthermia group. (F) Villanueva bone staining of the rat models at 2 weeks and 4 weeks. New mineralized bone was stained as a green area in the heating material (original magnification x20). Right column shows higher magnification (x 100, bars; 200μm) of middle column (x20, bars; 1mm). The areas of mineralized bone were measured at 2 (G) and 4 weeks (H). All data are expressed as the mean ±SD. *p<0.05 compared with control group.

**Fig 3:**
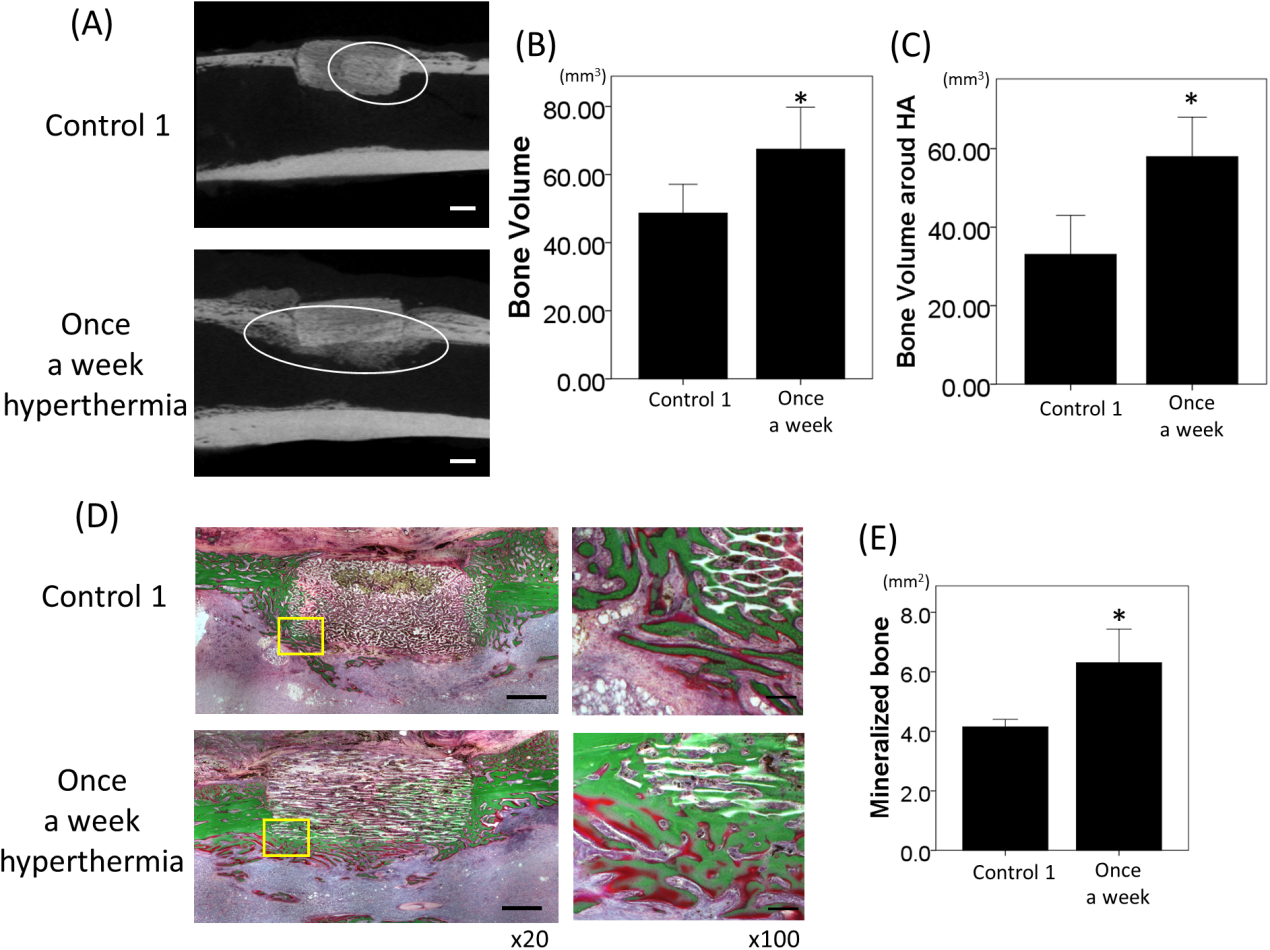
Radiographic and histological evaluation of hyperthermia in rabbit model. (A) Sagittal micro-CT image of the right tibia in control 1 and once a week hyperthermia at 4 weeks. White circling depict the newly formed bone in (B) and around (C) the grafted materials (control 1, once a week hyperthermia; n = 5 each) at 4 weeks (bars; 2mm). *p<0.05 compared with control 1 group. (D) Villanueva bone staining of the tibia of rabbit models in control 1 and once a week hyperthermia group at 4 weeks. Mineralized bone was stained as green and osteoid as red. Right column (magnification x100, bars; 200μm) depicts the higher magnification of left column (original magnification x20, bars; 2mm). (E) The areas of mineralized bone at 4 weeks was calculated and graphed. All data are expressed as the mean ±SD. *p<0.05 compared with control 1 group.

As compared to a previous hyperthermia study [29], in which alginate gel was used as a carrier for magnetite, the osteogenesis in the HA was observed in all specimens of rats and rabbits in a time-dependent manner. The new bone formation occurred in the HA, beginning from the periphery of HA where cortical and cancellous bone were in contact with the material in rats (Fig 2A). In contrast, osteogenesis was observed not only in the HA but also around the material in rabbit, particularly in the hyperthermia group (Fig 3A). In the rat model, once a week hyperthermia significantly increased new bone formation in the HA as compared with the control at both 2 and 4 weeks (p = 0.02 and p = 0.018, respectively) (Fig 2B and 2D). All control 1, 2, and 3 groups showed similar results, suggesting that HA with AMF could not effectively increase the temperature, and not stimulate osteogenesis. BMD of the HA was also significantly higher in the once a week hyperthermia group at both 2 and 4 weeks (p = 0.01 and p = 0.012, respectively) (Fig 2C and 2E). In contrast, new bone formation did not increase in rats of the three times a week hyperthermia group compared with those in the control group at either 2 or 4 weeks (Fig 2A). There was no significant difference in bone volume between the control and three times a week hyperthermia group (2W; p = 0.380, 4W; p = 0.115, respectively) (Fig 2B and 2D). BMD of grafted heating materials in the three times a week hyperthermia group was significantly decreased compared with that in the control group at 4 weeks (p = 0.015) (Fig 3E).

In the rabbit model, there was significantly increased new bone formation in the HA of the hyperthermia group compared with that in the control 1 group at 4 weeks (p = 0.036) (Fig 3B). Vigorous osteogenesis was also observed around the HA, adjacent area of bone marrow space and surrounding space of cortical bone in the hyperthermia group (Fig 3A). New bone formation around the HA was also set as ROI and evaluated. Bone volume around the HA was statistically greater in the hyperthermia group as compared with the control 1 group (p = 0.008) (Fig 3C).

### Histological evaluation of osteogenesis

**Fig 4:**
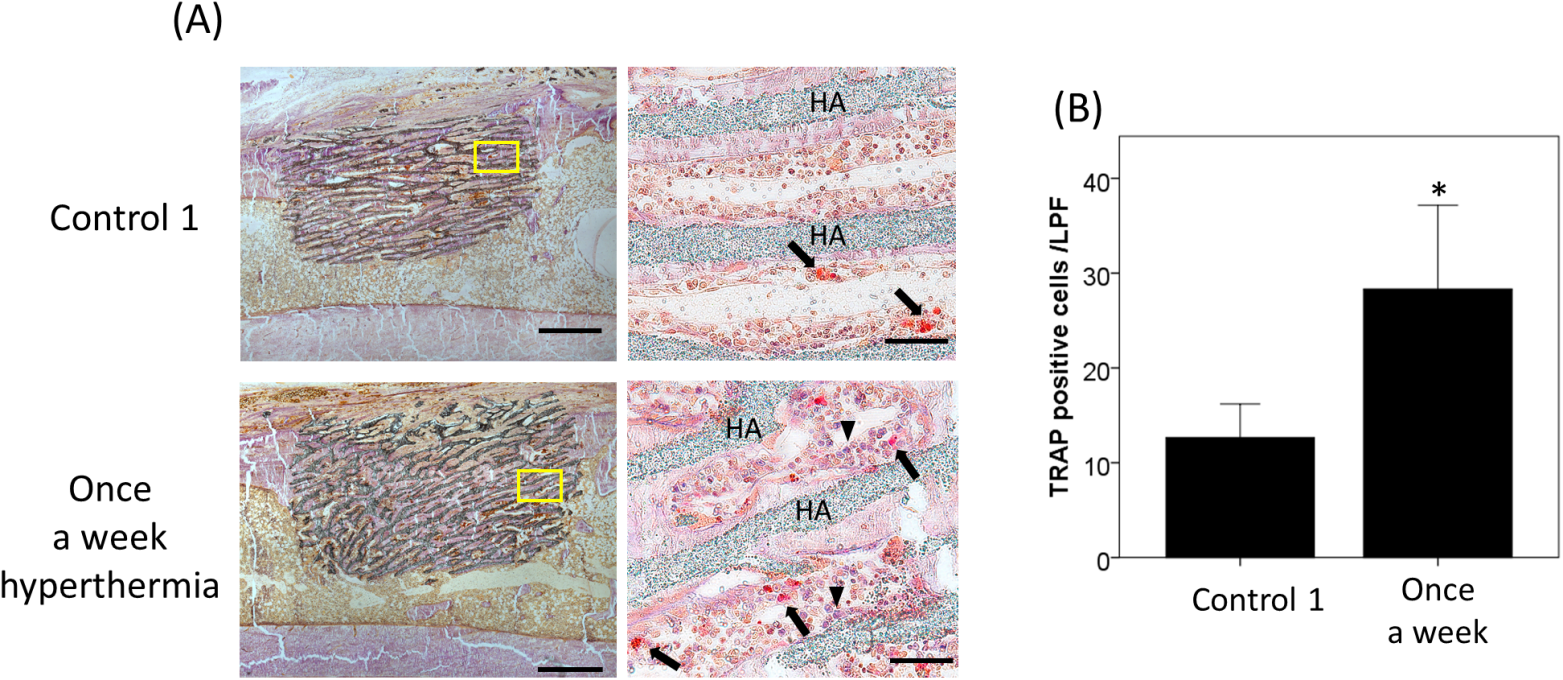
TRAP and ALP double staining at 4 weeks in the rat model. (A) Left column shows sagittal section of the right tibia in control 1 and once a week hyperthermia group (original magnification x20, bars; 1mm). Right column shows images of higher magnification (x200, bars; 100μm) of left yellow square. TRAP-positive red cells (arrows) and ALP positive purple cells (arrowheads) were observed around the newly formed bone. (B) TRAP-positive cells/ LPF in five different areas in hyperthermia and control 1 group were measured and graphed. Data are expressed as the mean ±SD. *p<0.05 compared with control 1 group. HA: (hydroxyapatite)

The effects of hyperthermia on new bone formation were evaluated microscopically by Villanueva bone staining in and around the grafted HA as well as radiographical evaluation with micro-CT. Calcified bone stained green was more widely observed in the once a week hyperthermia group compared to the control 1 group, particularly from the periphery of HA to the center along the unidirectional porous structures of the material in a time-dependent manner (Fig 2F). Quantification with Image J software revealed that the green area of the ROI in the once a week hyperthermia group was significantly increased compared with that in the control 1 group in both the 2- and 4-week rat models. (p = 0.036 and p = 0.026, respectively) (Fig 2F–2H). Mineralized bone colored in green in the rabbit model at 4 weeks was upregulated in the hyperthermia group not only in the grafted material but also surrounding area. Nonmineralized bone colored in red was observed in both groups, and the red-colored area was also increased in the hyperthermia group (Fig 3D). The area of mineralized bone in ROI in and around the grafted HA of rabbit was significantly higher in the hyperthermia group than control 1 group (p = 0.031, Fig 3E). The iron particles remained in the HA artificial bone even four weeks after implantation. Mineralized bone formation was poor in the three times a week hyperthermia group of rat, which was consistent with the results of the micro-CT analyses. In double staining of TRAP and ALP, TRAP-positive multinucleated cells were expressed around the newly formed bone (Fig 4A). Positive cell counting showed that significantly more TRAP-positive multinucleated cells were expressed in the once a week hyperthermia group than control 1 group (p = 0.020) (Fig 4B). ALP positive cells were observed on the suspected newly formed bone surface and around the junction of existing bone and heating material, whereas fewer ALP expressing cells were observed in the control 1 group.

### Effects of hyperthermia on osteogenic and chondrogenic differentiation

**Fig 5:**
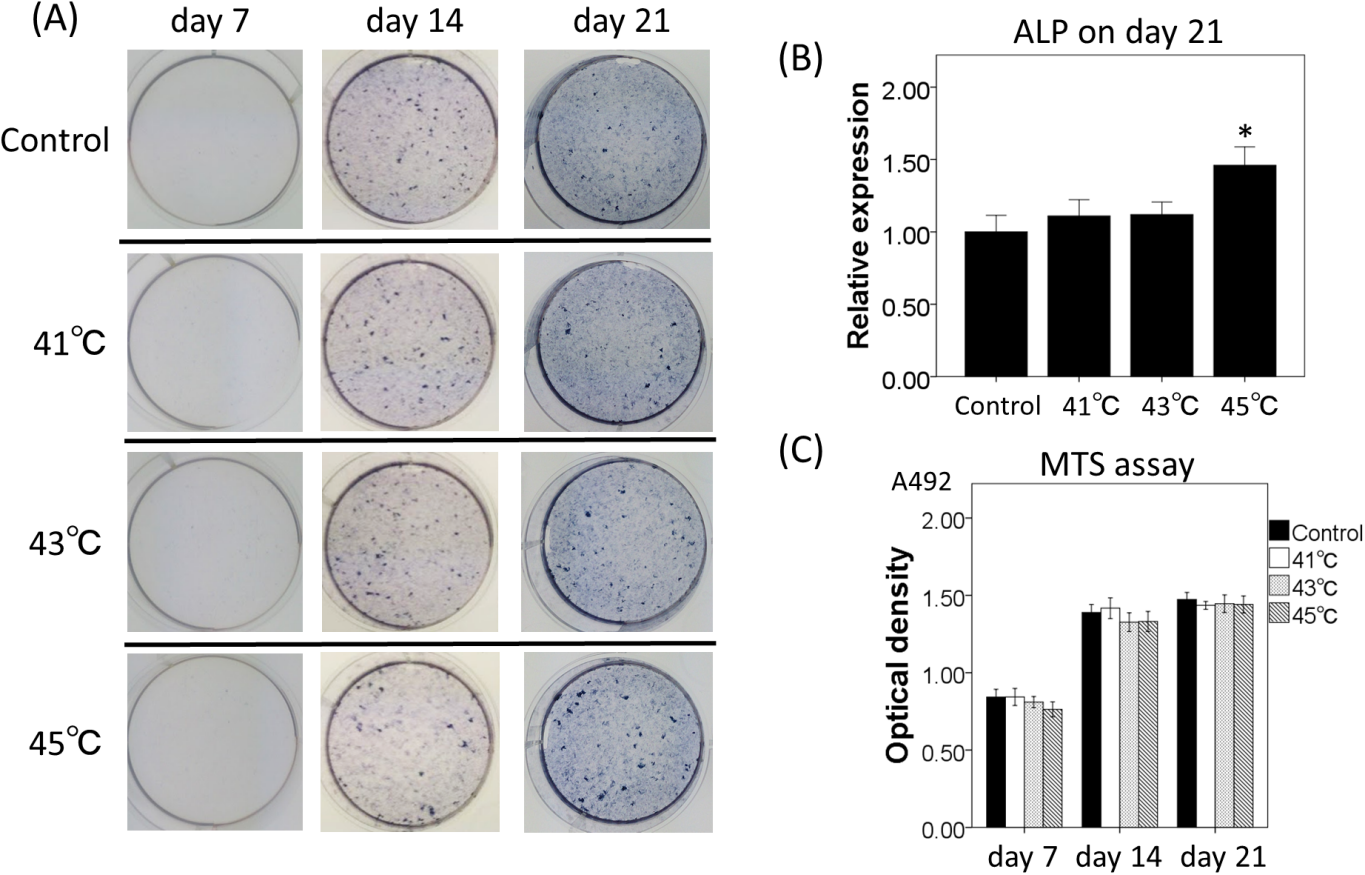
Effects of in vitro hyperthermia on differentiation and cell viability of MC3T3 cell line. (A) ALP staining of MC3T3 cells with or without treatment of hyperthermia (41, 43, and 45°C). Cultured wells were photographed at days 7, 14, and 21. ALP stained areas in the wells were measured by Image J software and graphed (B). Data are expressed as the mean ±SD. *p<0.05 compared with control group. (C) Effects of various temperatures on cell viability of MC3T3 cells measured by MTS assays on day 7, 14 and 21. Data are expressed as the mean ±SD.

**Fig 6:**
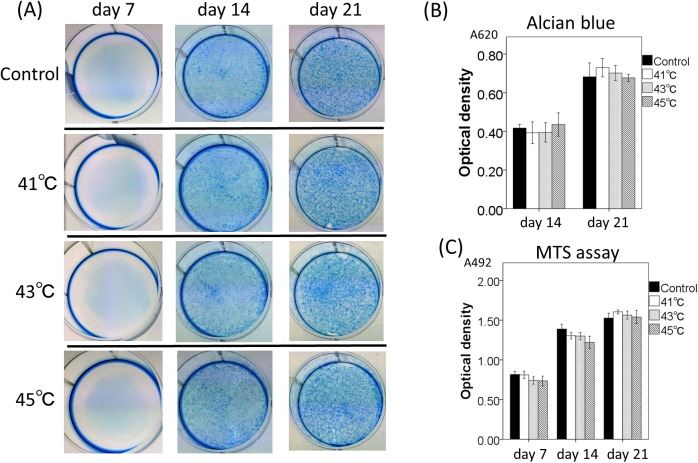
Effects of hyperthermia on differentiation and cell viability of ATDC5 cell line. (A) Alcian blue staining of ATDC5 cells with or without treatment of hyperthermia (41, 43, and 45°C). Cultured wells were photographed at days 7, 14, and 21. (B) The quantitative measurement of proteoglycan amount in cultured ATDC5 cells at days 14 and 21 with or without treatment of hyperthermia (41, 43, and 45°C). (C) Effects of various temperatures on cell viability of ATDC5 cells measured by MTS assays on day 7, 14 and 21. Data are expressed as the mean ±SD.

ALP staining for osteogenic cell line, MC3T3 cells, revealed that positive staining was microscopically confirmed as positive at day 7, and macroscopically positive at day 14 in all hyperthermia and control group cultures. (Fig 5A). Quantification by Image J software showed that osteogenic differentiation significantly increased in the heat exposure groups (41, 43, and 45°C) at day 21 as compared with control group (p = 0.03). ALP expression was highest in cultures of the 45°C heat exposure group compared to the control (p = 0.001) (Fig 5B). The results of alcian blue staining for evaluation of hyperthermia effects on chondrogenic differentiation of ATDC5 cells identified no significant changes between any of the temperature examined or control cultures at any time point (day7, 14, and 21) (Fig 6A and 6B). To assess the adverse effects of hyperthermia, cell viability of MC3T3 and ATDC5 cells was investigated with MTS assay. There was no significant change in cell viability until 3 weeks in any of the hyperthermia groups or control (Figs 5C and 6C). Although cell viability with higher temperatures (43 or 45°C) tended to show lower viability at day 7 and 14, it showed recovery at day 21.

## Discussion

The results of the present study demonstrated that hyperthermia with clinically applicable HA (REGENOS) and contrast agent (Resovist) composite under AMF effectively induces significantly greater new bone formation in both a rat and rabbit model. Our previous study used alginate gel as a carrier for magnetite liposome in a rat tibia defect model, and the results showed heat-induced new bone formation in the surrounding area, but not in the grafted materials [29]. HA has been widely used clinically for bone repair, bone augmentation and as a coating material for orthopedic implants [34,35], and its bio-affinity and osteoconductive ability have been verified. The results of the present study demonstrated that heat-triggered newly formed bone was prominently located in the grafted HA along the unidirectional porous structures, which has been reported to have high osseous conduction potential [36,37]. Resovist is a liquid that contains iron and can be heated with AMF. Sato et al. reported the feasibility of hyperthermia therapy using AMF for Resovist as a heating material [30]. Compared with HA, heat-triggered osteogenic precursor cells could not penetrate into the alginate gel used in the previous study, which did not show sufficient osteoconductive ability. Another difference from the previous study [29] was that the heat stimulus was repeatedly given in the present study (present study; once or three times a week, previous study; just once), and the temperature given was set at 45°C in the present study (previous study; 43–46°C). This temperature was chosen for the present study based on the previous results of radiographic evaluation at 2 weeks after hyperthermia ranging from 43–46°C, which showed that the effects were maximal at 45°C [29]. Although increased osteogenesis was observed at 2 weeks after treatment, the efficacy was found to decrease in a time-dependent manner in the previous study. The present study demonstrated that scheduled hyperthermia could continuously stimulate the osteogenesis, whereas excess treatment (three times a week) might reduce it. Regarding cellular stress responses to hyperthermia, Fulda et al. reported that the initial cellular response to a stressful stimulus is geared towards helping the cell to defend against and recover from the insult, but then if the noxious stimulus remains unresolved cells activate death signaling pathways [38]. Other studies pointed out that heat shock proteins (HSPs) are essential for such a reaction to thermal stress in various types of cell [39,40]. The rats subjected to 3 times a week heat stimulation tended to show reduced body weights (data not shown).

In the present study, micro-CT was used to evaluate osteogenesis in the HA. The soft X-rays examination used for evaluation in the previous study [29] was inadequate for the present study, in which radiopaque materials were used, and new bone formation was observed in the grafted materials. Examination with micro-CT also enabled the evaluation of patterns of bone formation, that is, from periphery of the materials and to the center.

An interesting finding in this study is that the patterns of bone formation differed between the rat and rabbit animal models. In rats, new bone appeared only in the grafted HA, whereas new bone was formed in and around the HA in rabbits. The review of animal models in fracture healing by Loughlin et al [41] described that ossification during fracture healing in higher animal species showed a healing pattern similar to that of humans with ossification typically manifest before cartilage is present and both processes coexisting in normal bone repair. However, in bone healing of rodents (rats and mice), endochondral bone formation predominates although the two processes of bone formation are present. Such differences in heat-triggered osteogenesis among animal species merit further investigation.

Several previous studies analyzed the in vitro thermal effects on osteogenesis using osteoblast-like MC3T3 cells. Dolan et al reported that MC3T3 cells exposed to severe heat shock over 47°C for 30 sec developed cell necrosis and apoptosis [26]. On the other hand, Kajiya et al showed that everyday thermal treatment at 42°C for 10min. increased the activity of ALP in MC3T3 cells in a time-dependent manner compared with the non-thermal stress control [42]. Chen et al demonstrated the upregulated osteoblastic differentiation from MSCs with mild heat shock (41°C) [19]. The results of cell viability experiments in the present study indicated that 45°C thermal treatment in vitro does not harm MC3T3 cells seriously, and stimulates ALP activity. Compared with the effects of osteogenesis in the previous study [42], those in the present study seemed to be weak. This is likely to be attributable to differences in temperature and the frequency of treatments between them. Thermal treatment at 45°C to ATDC5 cells could not alter the chondrogenic differentiation, suggesting that hyperthermia could not stimulate endochondral differentiation, but osteoblastic ossification. Together with the results of previous studies, heat-stimulus might induce new bone formation probably via osteoblastic ossification.

Mechanisms of enhanced osteogenesis by heat stress have not been clearly elucidated. Involvement of HSP70 in the promotion of osteogenesis has been reported using human MSCs [25]. HSP70 increases ALP activity, and promotes hMSC mineralization. HSP70 significantly upregulated the expression of osteo-specific genes including Runx2 and osterix. The present and our previous study [29] showed upregulation of ALP expression and TRAP positive cells in hyperthermia group, which is in consistent with the study by Chen et al [25]. Participation of HSPs, such as HSP70, in the present system should be analyzed further. As one of the other possible mechanism, Li et al. reported that heating led to the induction of angiogenesis [43], suggesting that angiogenesis within and around the grafted HA, which has osteoconduction ability, contributes to the osteogenesis.

There are several limitations in the present study. The amount of Resovist contained in the heating materials was the theoretical value. It may decrease during the preparation process and experimental course. Strict thermal control is crucial for the experiments and clinical application. Initially, increased temperatures were measured directly by the inserted probe, but considering the risk of infection, the overlying skin temperature was measured as an alternative thereafter. The Resovist within the grafted HA might be washed away over time, possibly reducing the efficacy of the hyperthermia in the long term. However, the results of the histological staining showed that the iron remained in the grafted HA at 4 weeks, while the skin temperature was effectively increased in the present study. Longer term investigations on Residual Resovist and thermal efficacy will be required. Second, although new bone formation induced by hyperthermia was demonstrated in the two species of animal in the present study, the patterns of induced osteogenesis may be different in larger animals including man. Moreover, because the amount of grafted HA required for larger animals would be greater, the thermal effects on osteogenesis will need to be investigated with use of a large amount of HA and Ferucarbotran composite under AMF. Third, the precise mechanisms for heat stimulus osteogenesis in vivo were not addressed or clarified. Detailed mechanism should be clarified in this heating system.

## Conclusions

We used clinically applicable materials, HA and Ferucarbotran composite, for hyperthermia in the rat and rabbit, and found that hyperthermia effectively induced significantly more newly formed bone in the defect. No severe side effects were apparent throughout the experiments. These results show that appropriate heat-stimuli with clinically applicable heating materials can promote enhanced osteogenesis, and that this procedure with combination materials may be a promising treatment option for bone defects in various skeletal diseases in the future.
